# Omega-3 polyunsaturated fatty acid-induced vasodilation in mouse aorta and mesenteric arteries is not mediated by ATP-sensitive potassium channels

**DOI:** 10.3389/fphys.2022.1033216

**Published:** 2022-12-15

**Authors:** Cristiana Bercea, Roshan Limbu, Kamila Behnam, Keat-Eng Ng, Qadeer Aziz, Andrew Tinker, Francesco Tamagnini, Graeme S Cottrell, Alister J McNeish

**Affiliations:** ^1^ McNeish Laboratory, School of Chemistry, Food and Pharmacy, Department of Pharmacology, University of Reading, London, United Kingdom; ^2^ Tinker Laboratory, William Harvey Research Institute, Clinical Pharmacology and Precision Medicine, Queen Mary University, London, United Kingdom

**Keywords:** omega-3 fatty-acids, KATP, DHA, EPA, PNU-37883A, potassium channels

## Abstract

There is strong evidence that the omega-3 polyunsaturated fatty acids (n-3 PUFAs) docosahexaenoic acid (DHA) and eicosapentaenoic acid (EPA) have cardioprotective effects. n-3 PUFAs cause vasodilation in hypertensive patients, in part controlled by increased membrane conductance to potassium. As K_ATP_ channels play a major role in vascular tone regulation and are involved in hypertension, we aimed to verify whether n-3 PUFA-mediated vasodilation involved the opening of K_ATP_ channels. We used a murine model in which the K_ATP_ channel pore subunit, Kir6.1, is deleted in vascular smooth muscle. The vasomotor response of preconstricted arteries to physiologically relevant concentrations of DHA and EPA was measured using wire myography, using the channel blocker PNU-37883A. The effect of n-3 PUFAs on potassium currents in wild-type native smooth muscle cells was investigated using whole-cell patch clamping. DHA and EPA induced vasodilation in mouse aorta and mesenteric arteries; relaxations in the aorta were sensitive to K_ATP_ blockade with PNU-37883A. Endothelium removal didn’t affect relaxation to EPA and caused a small but significant inhibition of relaxation to DHA. In the knock-out model, relaxations to DHA and EPA were unaffected by channel knockdown but were still inhibited by PNU-37883A, indicating that the action of PNU-37883A on relaxation may not reflect inhibition of K_ATP_. In native aortic smooth muscle cells DHA failed to activate K_ATP_ currents. We conclude that DHA and EPA cause vasodilation in mouse aorta and mesenteric arteries. Relaxations in blocker-treated arteries from knock-out mice demonstrate that K_ATP_ channels are not involved in the n-3 PUFA-induced relaxation.

## 1 Introduction

The cardioprotective effects of marine-derived omega-3 polyunsaturated fatty acids (n-3 PUFAs) first drew attention when epidemiological studies found a lower incidence of cardiovascular diseases in regions of the world with a high consumption of fish (Bang et al., 1980; Kagawa et al., 1982). Since then, numerous studies have confirmed that n-3 PUFAs have vasodilatory effects in hypertensive humans, as reviewed in (Miller et al., 2014; Colussi et al., 2017; AbuMweis et al., 2018), and in preclinical models of hypertension, as reviewed in (Mozaffarian and Wu, 2011; Bercea et al., 2021). Two of the most studied n-3 PUFAs involved in vasodilation are eicosapentaenoic acid (EPA) and docosahexaenoic acid (DHA); these are constituents of the lipid component of cell membranes, and are known to modulate the activity of ion channels, such as K^+^ channels (Hamilton and Kamp, 1999).

Hypertension is associated with endothelium and vascular smooth muscle cell (VSMC) dysfunction, reviewed in (Touyz et al., 2018). However, in blood vessels isolated from animals, the endothelium appears to play only a limited role in the short-term effects of DHA and EPA on vascular tension. For example, in rat aorta and mesenteric arteries, DHA and EPA-induced relaxation is only slightly reduced after endothelium removal (Engler and Engler, 2000; Engler et al., 2000; Sato et al., 2013; Sato et al., 2014; Limbu et al., 2018), and EPA-induced relaxation is reduced but not abolished in sheep pulmonary artery (Singh et al., 2010). Contraction in VSMCs is largely controlled through Ca^2+^ entry. This is opposed by K^+^ efflux leading to hyperpolarization, closure of Ca^2+^ channels, and vasodilation (Nelson and Quayle, 1995). Multiple VSMC mechanisms are proposed to explain the effect of n-3 PUFAs, many of which include the hyperpolarizing effects of activating K^+^ channels. Limited results were obtained with respect to L-type voltage-gated Ca^2+^ channel involvement in n-3 PUFA-mediated vasodilation (Engler and Engler, 2000; Engler et al., 2000; Singh et al., 2010), whereas K^+^ channels have been found to play a greater role.

K^+^ channels involved in the regulation of vascular tone have been found to be activated by n-3 PUFAs, reviewed in (Elinder and Liin, 2017), leading to vasodilation. Multiple studies have investigated the ion channel targets of n-3 PUFAs, and found only a small contribution from BK_Ca_ (Ye et al., 2002; Lai et al., 2009; Wang et al., 2011; Hoshi et al., 2013; Sato et al., 2014; Nagaraj et al., 2016) and no contribution from Kv1.2 or 1.3 (Sato et al., 2014), SK_Ca_ (Limbu et al., 2018) as well as other types of K_Ca_ (Engler and Engler, 2000; Engler et al., 2000; Villalpando et al., 2015), and IK (Sato et al., 2014) channels (Liin et al., 2015; Larsson et al., 2018).

Vascular K_ATP_ channels are involved in responses to vasodilators, which seem to stimulate these channels (Nelson et al., 1990; Kubo et al., 1997; Quayle et al., 1997; Quinn et al., 2004). Nitric oxide leads to hyperpolarization in rabbit mesenteric artery VSMCs by activating K_ATP_ channels (Murphy and Brayden, 1995), and prostanoids also activate K_ATP_ channels (Hide et al., 1995; Ney and Feelisch, 1995; Eguchi et al., 2007). Pharmacological agents used to study vasodilation have also been used to study these effects, for example channel openers like cromakalim, and blockers such as PNU-37883A (Humphrey et al., 1996). Clinically, openers of vascular K_ATP_ channels such as pinacidil can be used in the treatment of hypertension (Friedel and Brogden, 1990). Despite this, very few studies have investigated a direct role of K_ATP_ channels in n-3 PUFA-mediated vasodilation, but some have identified a contribution. For example, in rat aorta DHA and EPA (or their metabolites) induced vasodilation in an endothelium-independent manner, which is reduced by treatment with K_ATP_ channel blockers (Engler and Engler, 2000; Sato et al., 2013; Sato et al., 2014).

Vascular K_ATP_ channels are composed of a Ki6.1 pore subunit, and a SUR2B accessory subunit (Yamada et al., 1997). Global genetic deletion of either subunit leads to hypertension and coronary artery vasospasm, resulting in absence of hyperpolarising currents and death (Chutkow et al., 2002; Miki et al., 2002). Finally, knocking out K_ATP_ channel subunits in either the endothelium or VSMC has been suggested to have detrimental effects on coronary artery circulation (Kakkar et al., 2006; Malester et al., 2007; Aziz et al., 2014; Aziz et al., 2017).

In addition to the cell membrane, the inner membrane of mitochondria also contains K_ATP_ channels, which mediates ATP-sensitive K^+^ currents (Paggio et al., 2019). In most part, plasma membrane K_ATP_ and mitochondrial K_ATP_ are regulated by the same ligands (Paucek et al., 1992); moreover, pinacidil, a vasodilator, activates both types of channel to a similar extent (Liu et al., 2001). Mitochondrial K_ATP_ exhibits many properties similar to those of the plasma membrane K_ATP_ channel, for example the subunit structure appears to be qualitatively similar (Garlid et al., 1996; Bajgar et al., 2001). Loss of the mitochondrial Kir pore subunit suppressed activation by diazoxide, a K_ATP_ channel opener (Foster et al., 2012), and suppressed the cardioprotective effects of diazoxide (Paggio et al., 2019).

This study aimed to investigate whether concentrations of DHA and EPA relevant to those found in human plasma following meal enriched with these fatty acids (Newens et al., 2011) can activate vascular K_ATP_ channels leading to vasodilation. The actions of n-3 PUFAs have been found to be dependent on the type of artery (Shimokawa and Godo, 2016; Limbu et al., 2018), so we investigated both the aorta (elastic artery) and the mesenteric (resistance artery). Secondly, we studied whether n-3 PUFAs act through the pore subunit. For the latter, we used a mouse knock-out (KO) of VSMC Kir6.1; this KO leads to hypertensive animals and VSMC that do not respond well to vasodilators (Aziz et al., 2014).

## 2 Methods

### 2.1 Animals

All experiments were performed according to ARRIVE2.0 guidelines (Percie du Sert et al., 2020). Mice were maintained in 12 h:12 h light: dark cycle, a room temperature of 21°C and humidity of 50 ± 15%, with *ad libitum* access to food and water. Only male mice were used, as some studies have found that the blood pressure-reducing effects of DHA are stronger in men than women (Singhal et al., 2013). Moreover, there is evidence that acute supplementation of n-3 PUFAs does not affect outcomes related to post-prandial vasodilator responses in healthy female volunteers (Doppler flow and flow-mediated dilatation) but enhances them in males (Newens et al., 2011). 46 C57 BL6 mice were used housed at University of Reading, 25 C57 BL6 mice were used housed at William Harvey Institute Queen Mary University London, and 25 sm22cre + Kir6.1 (flx/flx) KO C57 BL6 mice were used housed at William Harvey Institute Queen Mary University London. The sm22cre + Kir6.1 (flx/flx) KO were obtained as discussed in (Aziz et al., 2014). Briefly, these are smooth muscle cell specific and obtained from crossing smooth muscle 22alpha promoter driven cre-transgenic mice (sm22cre) with Kir6.1 homozygous floxed (Kir6.1 (flx/flx)) mice, followed by a cross of the offspring. The KO animals were genotyped as previously described. All experiments were conducted in accordance with the Guide for the Care and Use of Laboratory Animals published by the British Home Office regulations (covered by project license PE9055EAD) and by the US National Institutes of Health (NIH Publication No. 85-23, revised 1996).

### 2.2 Materials

All salts for Krebs and electrophysiology solutions were obtained from Fisher Scientific. U46619 (Tocris BioTechne 1932; PubChem CID 5311493) and PNU-37883A (Tocris BioTechne 2095; PubChem CID 64392) were dissolved in 100% DMSO (PubChem CID 679), with further dilutions performed in DMSO; levcromakalim (Tocris 1378, PubChem CID 93504) was dissolved in distilled deionised water; DHA (Sigma D2534, PubChem CID 445580) and EPA (Sigma E2011, PubChem CID 446284) were dissolved in 100% ethanol, with further dilutions performed in distilled deionised water. The final concentration of ethanol was 0.3%. Phenylephrine (Tocris 2838, PubChem CID 6041) and acetylcholine (Sigma A6625, PubChem CID 6060) were dissolved in distilled deionised water. All stocks were prepared at 10 mM. MgATP (A9187, PubChem CID 5957) and NaADP (A2754, PubChem CID 6022) were purchased from Sigma.

### 2.3 Wire myography

8–10 week old male C57 BL6 mice were killed according to schedule one of the Animals (Scientific Procedures) Act 1986.

The animal was first anaesthetized using isoflurane, followed by immediate decapitation. The aorta and mesenteric vascular beds were extracted and placed on ice-cold Krebs buffer (118 mM NaCl, 3.6 mm KCl, 1.2 mm MgSO_4_.7H_2_O, 1.2 mm KH_2_PO_4_, 2.5 mm CaCl_2_, 11 mm glucose, 24 mm NaHCO_3)_.

The aorta and third order mesenteric artery (2 mm in length) were cleaned of fat and connective tissue and mounted on a wire myograph (Danish Myotechnology, 620M) connected to a force transducer (PowerLab ML846, ADInstruments) and the Labchart 7 software suite. The organ bath was filled with Krebs heated at 37°C and bubbled with carbogen (95% O_2_ and 5% CO_2_). The tissue was subjected to zero tension followed by equilibration for 20 min. The tissue was then stretched to a standardized tension of 5–6 mn (aorta) and 1-2 mn (mesenteric artery) according to the DMT normalization module in Labchart 7.

The presence of a functional endothelium was tested by first constricting the artery with 1–3 µm phenylephrine, followed by relaxation with 1 µm acetylcholine. A relaxation of over 50% was considered to represent a viable endothelium. In experiments investigating the role of the endothelium, the artery was tested as before, mechanically denuded by rubbing a 40 µm silver wire around the inner wall, and retested. A relaxation of less than 10% was considered to indicate a lack of functional endothelium.

After resting, the arteries were constricted with 10–30 nM U46619. The concentration response curves to DHA and EPA were then performed (for concentrations 100 nm–30 µm) in the presence or absence of drug treatment with 3 µm of the blocker PNU-37883A, which is purported to work through the Kir6.1 pore subunit of vascular K_ATP_ (Cui et al., 2003). For the aorta, a single U46619 concentration response curve was performed following 20 min treatment, with a time control aortic ring from the same aorta used as a control. For the mesenteric, two concentration response curves were performed in the same tissue, before and after treatment.

### 2.4 Isolation of aortic smooth muscle cells and patch-clamp electrophysiology

The aorta was collected as indicated above and placed in physiological solution (125 mm NaCl, 4.8 mm KCl, 1.2 mm KH_2_PO_4_, 1.1 mm EDTA, 1.7 mm MgCl_2_, 1 mm EGTA, 10 mm HEPES, and 11 mm glucose, pH 7.4). The artery was cleaned of fat and connective tissue, cut into four segments, and slit open. To isolate smooth muscle cells, the tissue was digested in low-Ca^2+^ physiological solution (136 mm NaCl, 5.6 mm KCl, 4.17 mm NaHCO_3_, 0.44 mm NaH_2_PO_4_, 0.42 mm Na_2_HPO_4_, 10.47 mm MgCl_2_, 0.1 mm CaCl_2_, 10 mm HEPES, pH 7.4), and then in dissociation solution (125 mm NaCl, 5 mM KCl, 0.1 mm CaCl_2_, 1 mm MgCl_2_, 10 mm NaHCO_3_, 0.5 mm KH_2_PO_4_, 0.5 mm NaH_2_PO_4_, 10 mm glucose, 10 mm HEPES, pH 7.2), as follows. The tissue was placed in low-Ca^2+^ physiological solution containing 2 mg/ml collagenase type 2 (Sigma 17101015) for 10 min at room temperature followed by 20 min at 37°C. The tissue was then washed in physiological solution and gently triturated. The tissue was then incubated in dissociation solution containing 0.7 mg/ml papain (Sigma P3125), 0.25 mg/ml BSA, and 0.5 mm DTT for 15 min at 37°C with shaking, and then in dissociation solution containing 0.5 mg/ml collagenase and 0.25 mg/ml BSA for 15 min at 37°C. The tissue was then washed in dissociation solution and triturated in dissociation solution. The cells were kept at 4°C until used. All recordings were made on the same days as the cell isolation.

K^+^ currents were recorded with a voltage clamp at −75 mV, in whole-cell configuration. These currents were evoked with a 30 m, 115 mV voltage step.

Whole cell currents were acquired with a Multiclamp 700B amplifier and digitised using a Digidata 1550B (Molecular Devices, United States), sampled at 100 kHz and lowpass filtered at 10 kHz. All data were visualized and stored onto a PC using the pClamp11 (Molecular Devices, United States) software routine.

The internal solution contained 107 mM KCl, 1.2 mM MgCl_2_, 1 mM CaCl_2_, 10 mm EGTA, 5 mm HEPES, 0.1 mm MgATP (Sigma A9187) and 1 mm NaADP (Sigma A2754), pH 7.2. The external solution contained 110 mm NaCl, 5 mm KCl, 1.2 mm MgCl_2_, 1.8 mm CaCl_2_, 10 mm glucose, and 10 mm HEPES, pH 7.2.

### 2.5 Data analysis and statistical procedures

Data analysis was performed in GraphPad Prism 7. Data is presented as mean ± SEM. We did not perform blinding or randomization.

For the analysis of myography data, and n of a minimum of five biological repeats were used, where n corresponds to the number of animals. Power calculations were conducted using the on-line tool http://www.stat.ubc.ca/∼rollin/stats/ssize/n2.html based on our preliminary myograph data. This study used the parameters of a common standard deviation, a normal distribution, a 0.05 type 1 error rate and a power of 80%. The vasodilatory response was calculated as a percentage of the reduction from the stable plateau of U46619 and the concentration response curve was plotted. To test for significance, two-way ANOVA multiple comparisons was used, followed by Bonferroni’s post-test. A *p*-value of <0.05 was considered statistically significant.

For the analysis of electrophysiological data, and n of eight biological repeats were used, where n is the number of animals from which cells were isolated or where cell lines were used form a separate passage. The current densities were calculated as a fraction of the baseline. We tested for normal distribution using the Shapiro-Wilk normality test, as it did not follow a normal distribution this data was presented as median and min to max whiskers. To test for significance, a Friedman test was performed. A *p*-value of less than 0.05 was considered statistically significant.

## 3 Results

### 3.1 The effect of n-3 PUFAs on mouse aorta and mesenteric arteries

#### 3.1.1 The role of K_ATP_ channels in n-3 PUFA-dependent relaxation in mouse aorta and mesenteric

**FIGURE 1:**
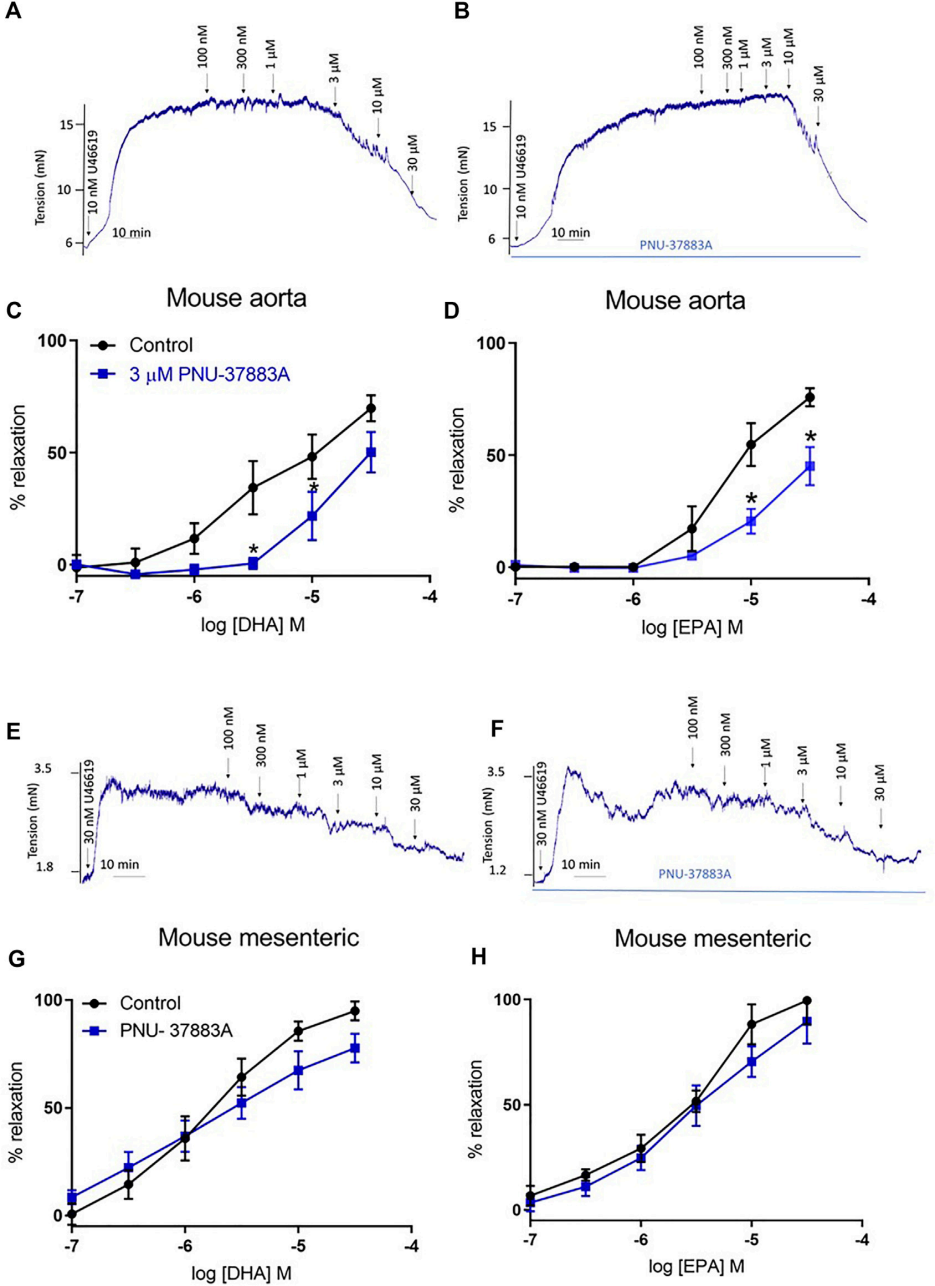
Concentration response curves showing the effects of inhibition of K_ATP_ channels on n-3 PUFA-induced relaxation in mouse aorta and mesenteric preconstricted with U46619. **(A)** Representative organ bath traces showing the effect of DHA (100 nm–30 μm) on mouse aortic tone. **(B)** Representative organ bath traces showing the effect of PNU-37883A treatment on DHA (100 nm–30 μm)-induced changes in mouse aortic tone. **(C)** DHA- and **(D)** EPA-induced relaxation in mouse aorta following treatment with the K_ATP_ inhibitor PNU-37883A (3 µm). **(E)** Representative organ bath traces showing the effect of DHA (100 nm–30 μm) on mouse mesenteric tone. **(F)** Representative organ bath traces showing the effect of PNU-37883A treatment on DHA (100 nm–30 μm)-induced changes in mouse mesenteric tone. **(G)** DHA- and **(H)** EPA-induced relaxation in mouse mesenteric following treatment with the K_ATP_ inhibitor PNU-37883A (3 µm). *n* = 5, data represented as mean ± SEM. *p < 0.05, significant difference from control curve assessed by 2-way ANOVA followed by Bonferroni multiple comparison test.

We studied the effects of DHA and EPA in mouse aorta and mesenteric arteries by conducting a concentration response curve to acute treatment following pre-constriction with U46619, a thromboxane A2 mimetic. Vehicle control experiments showed ethanol had no effect on tone (see representative experiment in Supplementary Figure S1). We did not use glibenclamide as a selective inhibitor in our studies as we (data not shown) and others (Cocks et al., 1990) have observed that it is an antagonist of thromboxane A2 receptors and leads to relaxation of U46619-induced tone. Comparable maximal tone was elicited in both treated and untreated vessels (Supplementary Figure S2). We found that in the aorta relaxation was significantly reduced for both EPA and DHA (Figures 1A–D), but that in the mesenteric artery there was no effect on n-3 PUFA-mediated relaxations (Figures 1E–H). Therefore, we continued further experiments only in the aorta.

#### 3.1.2 The role of the endothelium in n-3 PUFA-dependent relaxation in mouse aorta

**FIGURE 2:**
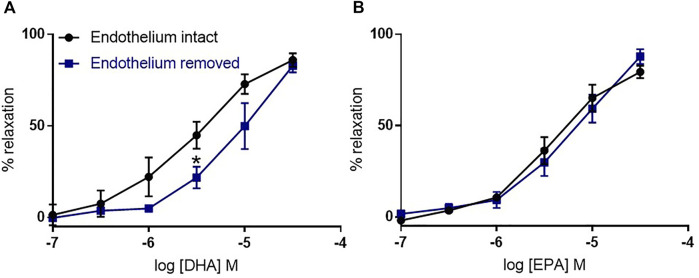
The effects of endothelium removal on DHA- and EPA-induced relaxation in mouse aortic rings preconstricted with U46619. **(A)** DHA- and **(B)** EPA-induced relaxation in denuded mouse aorta compared with intact rings from the same animal. *n* = 5, data represented as mean ± SEM. *p < 0.05, significant difference from control curve assessed by 2-way ANOVA followed by Bonferroni multiple comparison test.

We assessed the role of endothelium on n-3 PUFA-mediated dilations in the mouse aorta. We found that endothelium removal did not affect the relaxation to EPA, and partly reduced the relaxation to DHA at a low concentration (3 µm) (Figure 2).

### 3.2 Validation of Kir6.1 knock out model with an opener of K_ATP_ channels

**FIGURE 3:**
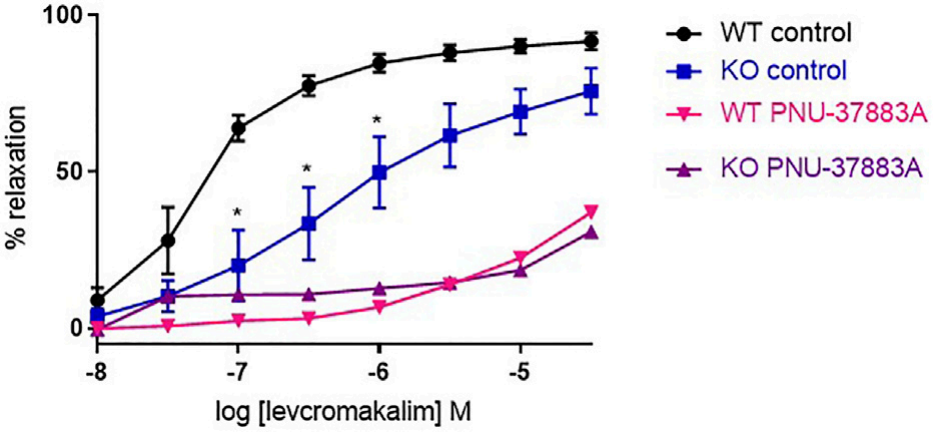
The effect of the K_ATP_-selective channel opener levcromakalim on Kir6.1 KO mouse aortic rings preconstricted with U46619. Levcromakalim-induced relaxation in WT and KO mouse aorta following treatment with the K_ATP_ inhibitor PNU-37883A (3 µm) compared with untreated rings from the same animal. *n* = 5, data represented as mean ± SEM. *p < 0.05, significant difference from control curve assessed by 2-way ANOVA followed by Bonferroni multiple comparison test.

We studied the role of the Kir6.1 subunit using a knock-out (KO) mouse model and compared this to wild-type littermate controls. We first confirmed that Kir6.1 KO channels do respond to K_ATP_-selective channel openers. To study this, we performed concentration response curves to the K_ATP_ opener levcromakalim, reported to act through the SUR subunit (Moreau et al., 2000). We found that levcromakalim causes relaxation in both the KO and the WT, with significantly less relaxation in the KO at low concentrations, and treatment with PNU-37883A inhibited relaxation in both WT and KO (Figure 3).

### 3.3 n-3 PUFA-dependent relaxation in Kir6.1 KO

**FIGURE 4:**
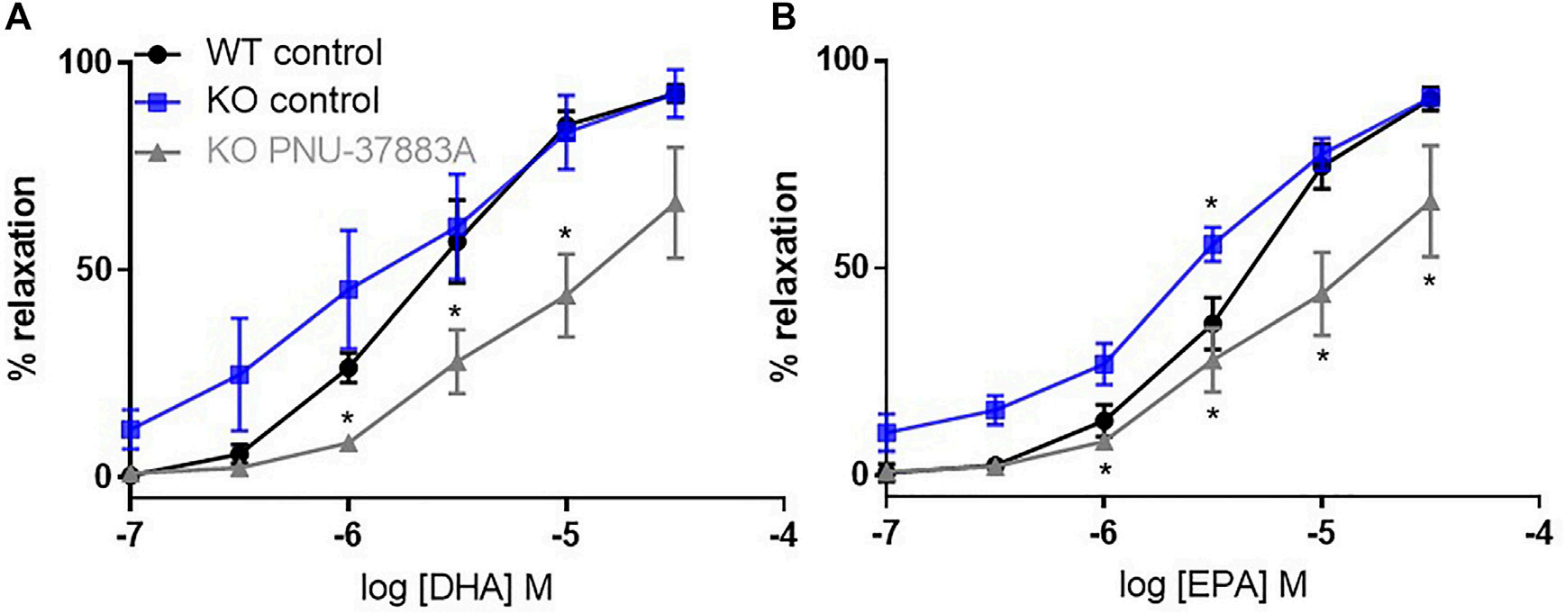
The effects of inhibition of K_ATP_ channels on n-3 PUFA-induced relaxation in Kir6.1 KO mouse aortic rings preconstricted with U46619. **(A)** DHA- and **(B)** EPA-induced relaxation in Kir6.1 KO mouse aorta following treatment with the K_ATP_ inhibitor PNU-37883A (3 µm) compared with untreated rings from the same animal. *n* = 5, data represented as mean ± SEM. *p < 0.05, significant difference from control curve assessed by 2-way ANOVA followed by Bonferroni multiple comparison test.

We then compared WT and KO responses to DHA and EPA (Figure 4). In the VSMC Kir6.1 KO model there was no reduction of the relaxation produced by DHA or EPA; interestingly, there appeared to be a small but significant increase in the relaxation produced. Thus, VSMC Kir6.1 does not appear to be involved in relaxations produced by n-3 PUFAs.

Surprisingly PNU-37883A led to a significant reduction in the percentage of relaxation to both DHA and EPA (Figure 4) despite Kir6.1 being the putative target of PNU-37883A.

### 3.4 The effect of DHA on K^+^ currents in vascular smooth muscle cells

**FIGURE 5:**
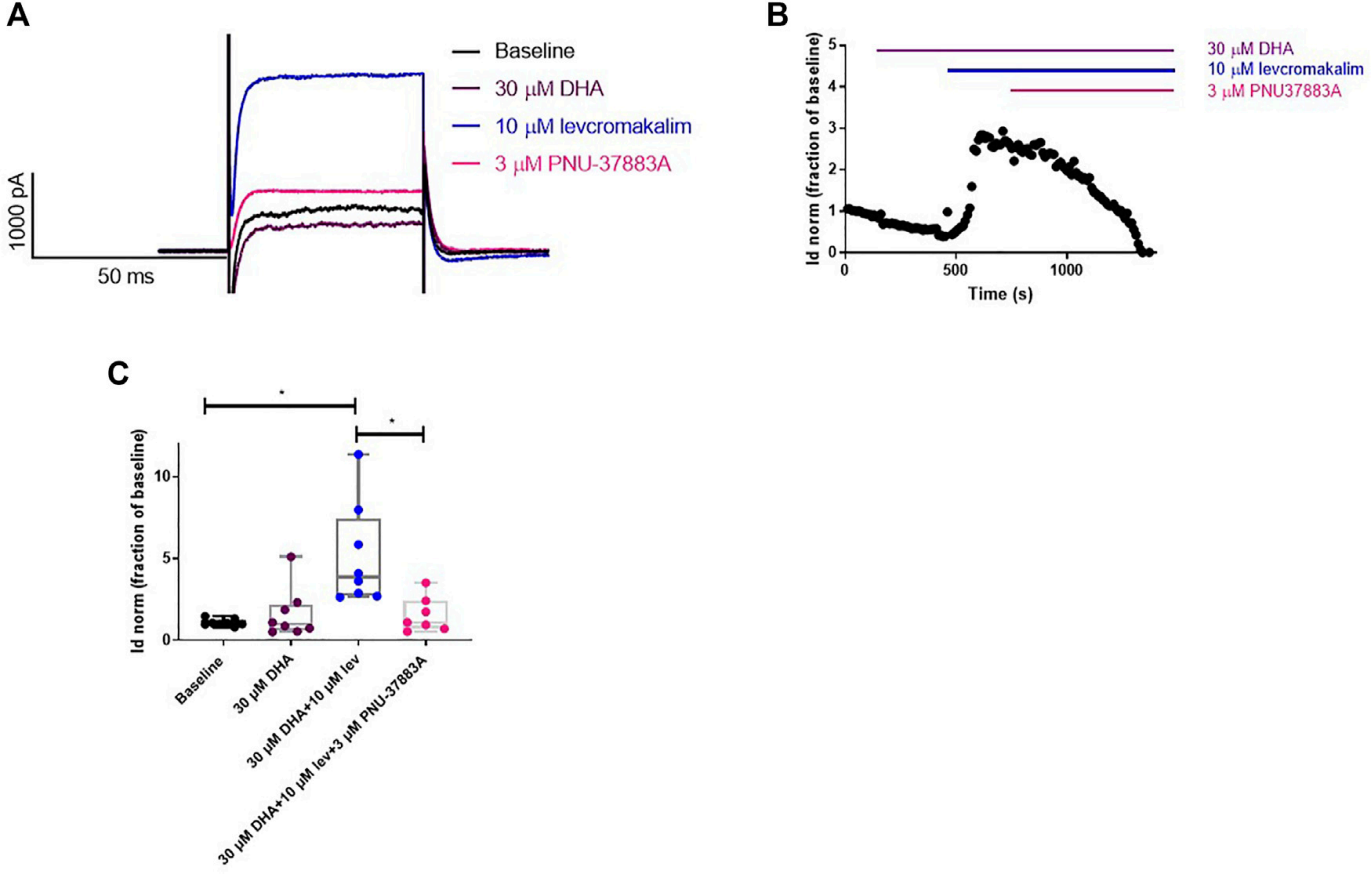
DHA does not affect K^+^ currents in vascular smooth muscle cells isolated from mouse aorta. **(A)** Representative current traces. **(B)** Representative whole cell current density time course trace. **(C)** DHA (30 µm) does not affect the K^+^ current density in primary mouse aorta VSMCs. The K_ATP_-selective opener levcromakalim (10 µm) causes an increase in these currents which is reversed by the K_ATP_ blocker PNU-37883A (3 µm). *n* = 8, data represented as normalised current density as a fraction of the baseline, with median and min to max whiskers. *p < 0.05, significant difference from baseline curve assessed by one-way ANOVA with Friedman’s test and Dunn’s correction for multiple comparisons. Lev = levcromakalim.

The effect of DHA on K^+^ currents in primary VSMC from WT mouse aorta was measured. We found that the bath application of 30 µm DHA had no effect on K^+^ current densities. However, the bath application of 10 µm levcromakalim resulted in increased current densities and this increase was reverted to baseline values by the subsequent bath application of PNU-37882A (Figure 5). These observations suggest that the currents measured from the primary aorta VSMCs contained a K_ATP_, levcromakalim-sensitive component and that DHA does not affect this component. We also found that currents in a stable cell line expressing the K_ATP_ structure Kir6.1/SUR2B were unaffected by DHA (Supplementary Figure S2), but activated by either levcromakalim or pinacidil, both of which were subsequently inhibited by PNU-37883A.

### 3.5 No role for mitochondrial K_ATP_ channels in n-3 PUFA-dependent relaxation in mouse aorta

**FIGURE 6:**
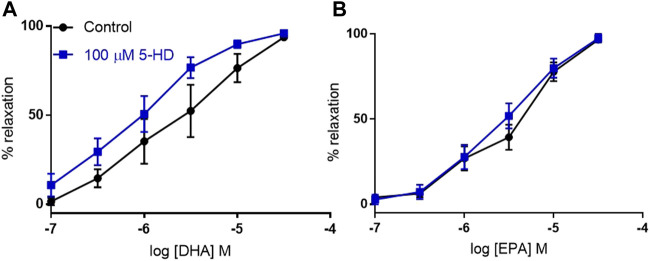
The effects of inhibition of mitochondrial K_ATP_ channels on n-3 PUFA-induced relaxation in mouse aortic rings preconstricted with U46619. **(A)** DHA- and **(B)** EPA-induced relaxation in mouse aorta following treatment with the selective mitochondrial K_ATP_ inhibitor 5-HD (100 µM) compared with untreated rings from the same animal. *n* = 5, data represented as mean ± SEM. *p < 0.05, significant difference from control curve assessed by two way ANOVA followed by Bonferroni multiple comparison test.

The Kir6.1 KO is expected to affect K_ATP_ in the VSMC plasma membrane, but it has not been established what the effect is on other K_ATP_ channels in VSMCs. We used the blocker 5-HD, which is proposed to act through the SUR subunit (Jaburek et al., 1998). We found that inhibition of mitochondrial K_ATP_ did not significantly affect relaxation to either DHA or EPA, confirming that mitochondrial K_ATP_ is not involved in the effect (Figure 6).

## 4 Discussion and conclusion

In this study we demonstrated using a sm22cre + Kir6.1 (flx/flx) Kir6.1 KO that it is highly unlikely that the subtype of K_ATP_ most common in smooth muscle cells (Kir6.1/SUR2B) underlies the n-3 PUFA-mediated relaxations observed in the aorta. This is despite relaxations produced by DHA and EPA having a major component sensitive to pharmacological blockade of K_ATP_ in WT mice. The results also call into question the selectivity of a pharmacological tool commonly used to inhibit K_ATP_ mediated responses.

K_ATP_ channels have been previously implicated in n-3 PUFA-mediated vasodilation, with K_ATP_ inhibition suppressing DHA-induced relaxation (Engler and Engler, 2000; Engler et al., 2000; Sato et al., 2013; Sato et al., 2014; Villalpando et al., 2015). Therefore, in this study we investigated whether DHA- and EPA-induced relaxations may involve these channels using pharmacological inhibitors. The actions of n-3 PUFAs show heterogeneity based on the type of artery (Shimokawa and Godo, 2016; Limbu et al., 2018), so we studied n-3 PUFA-mediated relaxation in both the aorta (elastic artery) and the mesenteric artery (resistance artery). We demonstrated that DHA and EPA caused concentration-dependent relaxations in both artery types at physiologically relevant concentrations (100 nm-30 µm) that are below the peak concentration recorded in human plasma after an n-3 PUFA rich meal (70 µm) (Newens et al., 2011). Relaxations in the aorta were largely endothelium-independent and significantly attenuated by the K_ATP_ blocker PNU-37883A but those in the mesenteric were unaffected.

Vascular K_ATP_ channels are composed of a Kir6.1 pore subunit and a SUR2B accessory subunit (Yamada et al., 1997). We investigated whether DHA and EPA activate vascular K_ATP_ channels using a mouse KO model of VSMC Kir6.1. We first confirmed Kir6.1 KO channel response to K_ATP_-selective channel opener levcromakalim, with levcromakalim being purported to activate K_ATP_ by an action on the SUR subunit (Schwanstecher et al., 1998; Hambrock et al., 1999). As expected, levcromakalim caused relaxation in both the KO and the WT, with significant inhibition of relaxation in the KO. The vascular K_ATP_ channel blocker PNU-37883A (Humphrey et al., 1996) inhibited levcromakalim-mediated relaxation in the KO, to the same degree as in the WT. The residual relaxation to levcromakalim may have been expected as Kir6.1/SUR2B K_ATP_ channel subtypes are expressed in the endothelium (Tang et al., 2010; Aziz et al., 2017). In addition, PNU-37883A may not only act through the Kir6.1 subunit; it might be inhibiting another channel such as a voltage-gated Ca^2+^ channels (Tomoda et al., 2005). We would have used another K_ATP_ blocker to confirm this, but our studies (data not shown) and others (Cocks et al., 1990) have shown that glibenclamide leads to relaxation of U46619-induced tone.

Having confirmed a role for VSMC K_ATP_ with the structure Kir6.1/SUR2B in vascular relaxation to levcromakalim, we compared WT and KO response to DHA and EPA and found no inhibition of the relaxation in the KO. Despite this, PNU-37883A still blocked this largely endothelium-independent relaxation, further indicating the inhibition of n-3 PUFA-mediated relaxation produced by PNU-37883A is highly unlikely to be due to an effect on K_ATP_ channels with subunit composition of Kir6.1/SUR2B. We considered whether the blocker might be inhibiting n-3 PUFA-mediated relaxation through the vascular subtype Kir6.2/SUR2B (Cui et al., 2003; Kovalev et al., 2004; Teramoto, 2006). However, this is unlikely as although this subtype mRNA is present in the KO, the channel is not functional (Aziz et al., 2014). A possible target is the endothelial Kir6.1/SUR2B subtype (Aziz et al., 2017), but this is unlikely because the relaxation to n-3 PUFAs that we observed is largely endothelium-independent.

The KO is expected to affect the pore subunit of plasma membrane vascular K_ATP_, but it has not been established what the effect is on other K_ATP_ channels in VSMC, such as mitochondrial K_ATP_. Mitochondrial K_ATP_ channels mediate ATP-sensitive K^+^ currents (Paggio et al., 2019), and are largely regulated by the same ligands as plasma membrane K_ATP_ channels (Paucek et al., 1992); the vasodilator pinacidil activates both types of channel to a similar extent (Liu et al., 2001). Moreover, mitochondrial K_ATP_ channels are activated by diazoxide, a K_ATP_ channel opener (Foster et al., 2012) which has cardioprotective effects (Paggio et al., 2019). In the present study, pharmacological blockade of mitochondrial K_ATP_ with 5-HD (Jaburek et al., 1998) did not significantly affect relaxation to either DHA or EPA, suggesting that mitochondrial K_ATP_ is not involved in this relaxation. This finding also eliminates the possibility that PNU-37883A might be acting on mitochondrial K_ATP_ channels.

As the myography data from the KO indicated n-3 PUFAs do not affect K_ATP_ currents and this was confirmed using whole cell patch clamp. DHA had no effect on the density of total K^+^ currents in native VSMC or stable cell lines expressing Kir6.1/SUR2B. This agrees with a previous study where a DHA-induced increase in outward K^+^ current in rat coronary VSMCs was not reduced by K_ATP_ channel inhibition with glyburide (Wang et al., 2011). These data confirm that the relaxation produced by n-3 PUFAs does not involve activation of vascular K_ATP_ (Kir 6.1/SUR2B) or any other K_ATP_ subtype.

One limitation of this study is that we did not investigate the contribution of other K^+^ channels. Of interest, several other types of potassium channel have been proposed to be involved in omega-3-mediated vasodilation, for example BK_Ca_ and Kv7.4/5 (Bercea et al., 2021). Notably, (Frampton et al., 2022), have recently demonstrated in *Xenopus* oocytes that n-3 PUFAs target the Kv7.4 and Kv7.5 subtypes, which are also highly expressed in vascular tissue. Therefore, future research should be focused on these channel subtypes in the vasculature.

In conclusion, this study found that DHA and EPA act to regulate vasodilation and hence are likely to reduce blood pressure, and these relaxations have a component apparently sensitive to the blockade of K_ATP_ in the aorta. However, knocking out the vascular smooth muscle cell pore subunit Kir6.1 had no effect on these vasodilatory responses, yet the putative Kir6.1 selective blocker PNU-37883A still led to a significant reduction in the percentage of relaxation to both DHA and EPA in the KO. Hence, the knock-out model data also casts light on the selectivity of PNU-37883A for VSMC K_ATP_ channels with the composition Kir6.1/SUR2B. We confirmed that n-3 PUFAs do not affect vascular K_ATP_ currents using whole-cell patch-clamping in native cells from WT mouse aorta, where DHA had no effect on the density of total K^+^ currents. This study provides evidence of heterogeneity between mouse aorta and mesenteric in the mechanisms of n-3 PUFA-mediated vasodilation and shows that plasma membrane and mitochondrial K_ATP_ do not mediate DHA or EPA-induced vasodilation.

## Data Availability

The raw data supporting the conclusions of this article will be made available by the authors, without undue reservation.
