# Supplementary material for: Omega-3 polyunsaturated fatty acid-induced vasodilation in mouse aorta and mesenteric arteries is not mediated by ATP-sensitive potassium channels
Source: Front Physiol. 2022 Dec 15;13:1033216. doi: 10.3389/fphys.2022.1033216 (PMC9797959; doi:10.3389/fphys.2022.1033216)
Supplement: Supplementary file 1 [file DataSheet1.PDF]

# *Supplementary Material*

## 1 The effect of vehicle control on mouse aorta preconstricted with U46619

We studied the effects of 0.3% of the vehicle control ethanol in mouse aorta following pre-constriction with U46619, a thromboxane A2 mimetic. Vehicle control experiments showed no effect of ethanol on tone.

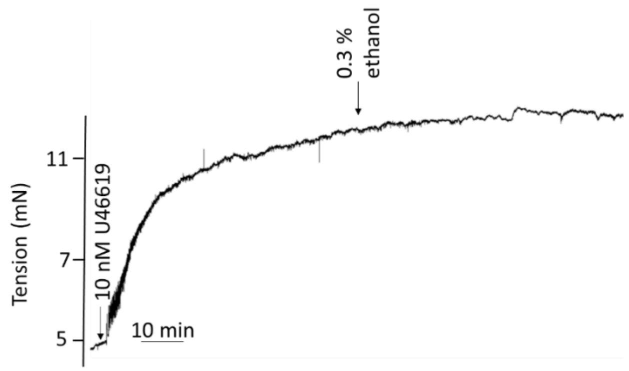


**Supplemental figure 1 The effect of vehicle control on mouse aorta preconstricted with U46619.** Representative organ bath traces showing the effect of ethanol (0.3%) on mouse aortic tone.

## 2 Constriction to U46619 in control and PNU-37883A-treated aortic rings

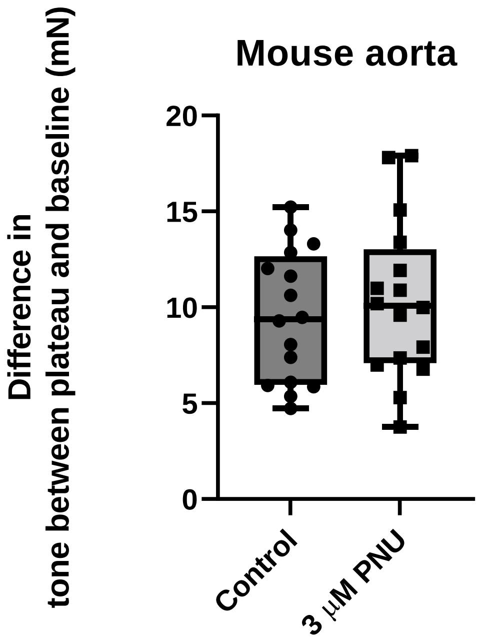


**Supplemental figure 2 Maximal tone elicited by U46619 in mouse aortic rings treated with PNU-37883A or untreated.** N=16, data represented as minimum to maximum values and mean ± SEM. *p<0.05, significant difference assessed by paired t-test.

## 3 The effect of DHA on $K^+$ currents in stable cell lines expressing $K_{ATP}$ channels

### 3.1 Materials and methods

### 3.2 Generation of HEK Flp-In-293 $K_{ATP}$ cell lines

HEK FlpIn 293 cells were cultured AT 37°C in a humidified atmosphere with 5% $CO_2$ in DMEM with 4.5g/L glucose L-glutamine and sodium pyruvate (Corning 10-013-CV) supplemented with 10% fetal bovine serum (Sigma F2442) and 100 µg/ml zeocin (Invivogen at-zn-05). Cells were transfected and stable cell lines generated expressing SUR2B/Kir6.1 in a two-step process where in the first step pcDNA5/FRT/SUR2B was transfected, followed by lentiviral transduction of pLenti6.3/FRT/Kir6.1.

In the first step, cells were transfected using Lipofectamine2000 (Invitrogen 11668030) according to manufacturer instructions using a transfection mix of 9:1 pOG44:plasmid DNA. Cells were selected using medium containing 200 µg/ml hygromycin and expression of SUR2B was verified using western blotting and immunocytochemistry.

In the second step, lentiviral particles containing Kir6.1 were produced in HEK Lenti-X 293 cells. Cells were transfected with viral plasmids, and lentiviral particles were purified after 48 hours using ViraBind lentivirus purification kit (Cell Biolabs VPK-104) according to manufacturer instructions. For the viral transduction, stable HEK FlpIn 293 cell lines that were initially transfected with pcDNA5/FRT/SUR2B were virally transduced with the purified lentiviral particles. Cells were then

selected using 200 µg/ml of hygromycin and 10 µg/ml of blasticidin. Expression of Kir6.1 and SUR2B in the same cells was then confirmed using Western blotting and immunocytochemistry.

## 3.3 Reagents, drugs, chemicals and other materials

All salts were obtained from Fisher Scientific. PNU-37883A (Tocris 2095) was dissolved in 100% DMSO, with further dilutions performed in DMSO; levcromakalim (Tocris 1378) was dissolved in in distilled deionised water; pinacidil (Sigma P154) ) was dissolved in 100% DMSO, with further dilutions performed in DMSO; DHA (Sigma D2534) was dissolved in 100% ethanol, with further dilutions performed in distilled deionised water. MgATP (A9187) and NaADP (A2754) were purchased from Sigma.

## 3.4 Electrophysiology

Whole-cell patch–clamp recordings were performed on the SUR2B/Kir6.1 stable cell line using the MultiClamp 700B amplifier (Molecular Devices). Signals were filtered at 2 kHz and sampled at 10 kHz using the Axon Digidata 1550B (Molecular Devices). Currents were recorded using a ramp protocol from -150 to +50 mV over 1 second with a holding potential of -80 mV with pClamp 10.6.

The intracellular solution contained 110 mM KCl, 1 mM $MgCl_2 6H_2O$, 10 mM EGTA, 10 mM HEPES, 3 mM MgATP, 1 mM NaADP. The extracellular solution contained 4 mM KCl, 1 mM $MgCl_2 6H_2O$, 130 mM NaCl, 10 mM HEPES, 10 mM D-glucose, 1.8 mM $CaCl_2$.

## 3.5 Data analysis

The mean current density at peak current values at +40 mV was calculated. No statistical analysis was performed on the data as n was lower than 5.

## 3.6 Results

We measured the effect of DHA on outward, whole-cell, $K^+$ currents in HEK293 stable cell lines expressing $K_{ATP}$ channels with the composition SUR2B/Kir6.1 (Supplemental Figure 1). We found that the bath application of 30 µM DHA has no effect on $K^+$ current densities. However, the bath application of 10 µM levcromakalim or 10 µM pinacidil, both openers of $K_{ATP}$ channels, resulted in increased current densities and such increase was reverted by the batch application of 3 µM PNU-37882A. These observations suggest that the currents contained a $K_{ATP}$, levcromakalim- and pinacidil- sensitive component and that DHA does not affect this component.

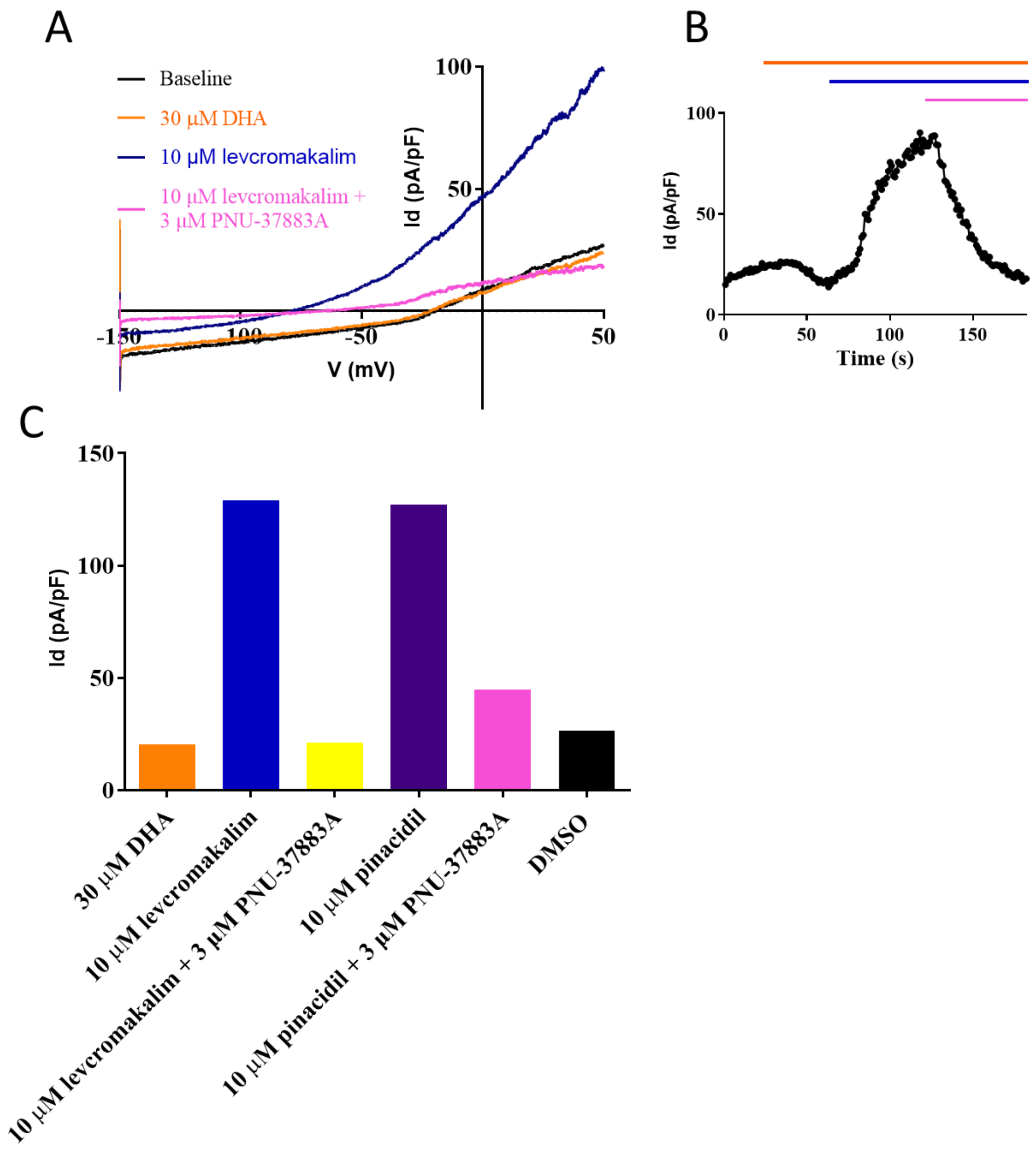


**Supplemental Figure 2 DHA does not affect K$^+$ currents in stable cell lines expressing K$_{ATP}$.** (A) Whole cell current density-voltage traces and (B) representative current density time course trace at +40 mV. (C) Mean current densities at +40 mV. Values were taken from the peak current obtained following application of DMSO (0.3 %) (n=4), levcromakalim (10 µM) (n=2), levcromakalim (10µM) + PNU-37883A (3 µM) (n=2), pinacidil (10 µM) (n=4), pinacidil (10 µM) + PNU-37883A (3 µM) (n=4) and DHA (30 µM) (n=4). The K$_{ATP}$-selective openers levcromakalim (10 µM) or pinacidil (10 µM) cause an increase in these currents which is reversed by the K$_{ATP}$ blocker PNU-37883A (3 µM).
